# Efficient and Selective Photogeneration of Stable N-Centered Radicals via Controllable Charge Carrier Imbalance in Cesium Lead Halide Nanocrystals

**DOI:** 10.1021/jacs.3c05323

**Published:** 2023-07-20

**Authors:** Tian Qiao, Madison E. Edwards, Xueting Tang, Xin Yan, Dong Hee Son

**Affiliations:** † Department of Chemistry, Texas A&M University, College Station, Texas 77843, United States; ‡ Center for Nanomedicine, Institute for Basic Science and Graduate Program of Nano Biomedical Engineering, Yonsei University, Seoul 03722, Republic of Korea

## Abstract

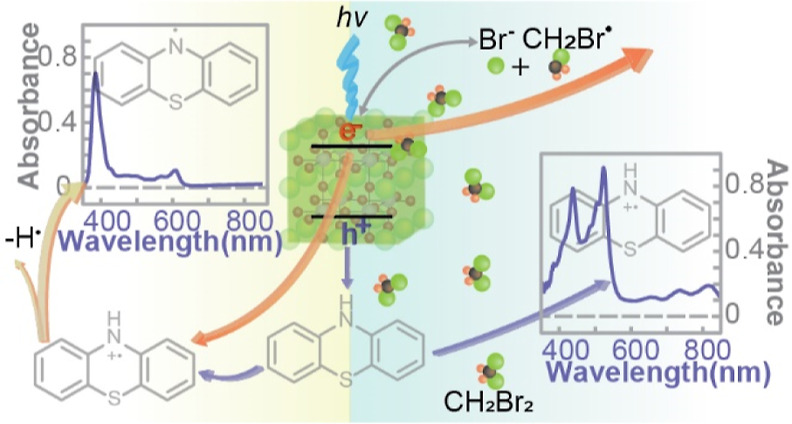

Despite the versatility of semiconductor nanocrystals (NCs) in photoinduced chemical processes, the generation of stable radicals has been more challenging due to reverse charge transfer or charge recombination even in the presence of sacrificial charge acceptors. Here, we show that cesium lead halide (CsPbX_3_) NCs can selectively photogenerate either aminium or aminyl radicals from amines, taking advantage of the controllable imbalance of the electron and hole populations achieved by varying the solvent composition. Using dihalomethane as the solvent, irreversible removal of the electrons from CsPbX_3_ NCs enabled by the photoinduced halide exchange between the NCs and the dihalomethane resulted in efficient oxidative generation of the aminium radical. In the absence of dihalomethane in solvent, the availability of both electrons and holes resulted in the production of an aminyl radical via sequential hole transfer and reductive N–H bond dissociation. The negative charge of the halide ions on the NC’s lattice surface appears to facilitate the aminyl radical production, competing favorably with the reversible charge transfer reverting to the reactant.

## Introduction

Photoinduced charge transfer using semiconductor nanocrystals (NCs) as the source of electrons and holes has been extensively utilized for various photochemical reactions including water splitting,^1–4^ CO_2_ reduction,^5–10^ decomposition of dyes,^11,12^ and organic coupling reactions.^13–18^ While semiconductor NCs can produce various species from the transfer of electrons or holes to the reactants, the generation of stable and persistent radicals via photoinduced charge transfer has been more challenging. Radicals are typically produced from closed-shell precursors via charge transfer and bond dissociation. Charge transfer from molecular redox photocatalysts such as metal complexes^19,20^ or the electrochemical cell^21^ is a common approach widely used in addition to the traditional chemical methods. When metal complex photocatalysts are used for radical production via charge transfer, back charge transfer that prohibits the effective formation of radicals is suppressed by modifying the ligand or by introducing irreversible downstream reactions.^22,23^ In principle, semiconductor NCs can also perform the photoinduced charge transfer necessary to convert the precursors to radicals reductively or oxidatively. However, the problems arising from back charge transfer or charge recombination are more difficult to address in semiconductor NCs using the same approaches. Although such problems can be partially mitigated by using sacrificial charge acceptors,^24^ reversible charge transfer cannot be completely blocked, often resulting in the generation of radicals only transiently, even for the species known to be stable in the absence of reversible charge transfer.

**Scheme 1:**
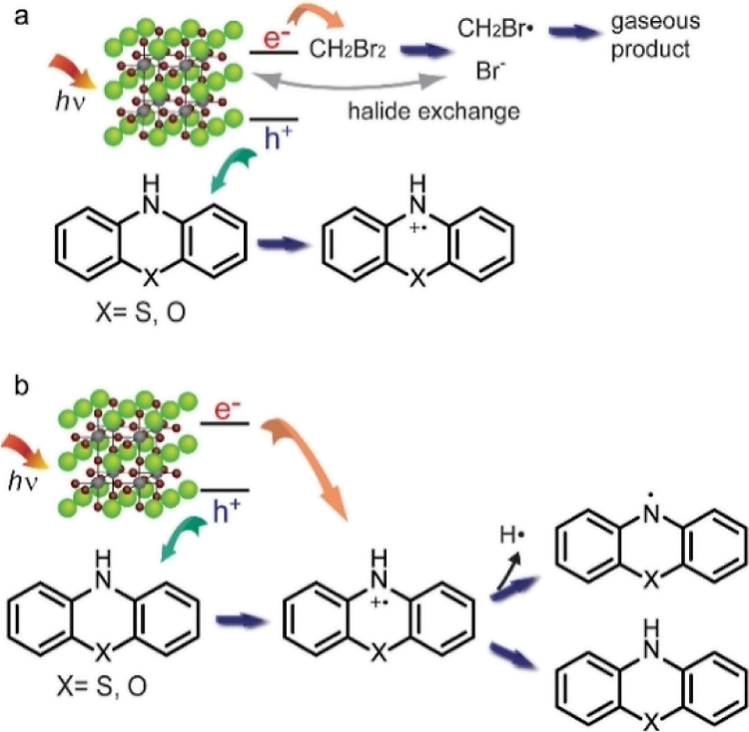
(a) One-Electron Oxidation of Secondary Aryl Amine by CsPbBr_3_ NCs in a CH_2_Br_2_/Hexane Mixture Forming Aminium Radical; (b) One-Electron Oxidation and Reductive Deprotonation of Secondary Aryl Amine by CsPbBr_3_ NCs in a Hexane Forming Aminyl Radical

Here, we report a perovskite NC-based method that effectively prevents the unproductive reversible charge transfer, enabling the efficient generation of an N-centered cation (aminium) or a neutral (aminyl) radical in a selective manner stably in solution and also performing radical–radical coupling reaction for a more reactive pair of radicals. The new method utilizes the unique ability of cesium lead halide (CsPbX_3_) NCs to control the population imbalance of the electrons and holes transferring to the precursor to perform either fully oxidative or redox-neutral radical formation, as illustrated in Scheme 1. The control of the electron and hole population imbalance is achieved via the photoinduced halide exchange reaction between the CsPbX_3_ NCs and the dihalomethane (CH_2_X_2_) solvent,^25^ where the exchanging X^–^ comes from the reductive dissociation of CH_2_X_2_ by the NCs. The halide exchange process is essential for irreversibly removing the electrons transferring from the NCs to CH_2_X_2_, which enables exclusive oxidative radical formation. When both electrons and holes are available from the NCs in the absence of CH_2_X_2_, a redox-neutral aminyl radical was formed stably via hole transfer, followed by reductive N–H bond breaking. The presence of the negative charge on the lattice surface, provided by halide ions, seems to assist the formation of aminyl radicals competing with the net null reaction resulting from the reversible charge transfer. This is an important advantage over other semiconductor NC photocatalysts such as II–VI quantum dots^26–28^ that exhibit a more limited capability to produce a stable population of aminium and aminyl radicals as compared in this work. The coupling reactions between the photogenerated radicals are also demonstrated, where the reaction benefits from a controllable charge carrier imbalance. We anticipate that the capability of CsPbX_3_ NCs demonstrated here can be expanded to other radical precursors, and the NC’s function can be enhanced by varying the lattice and ligand structure.

## Results and Discussion

To investigate the capability of CsPbX_3_ NCs to effectively produce and accumulate organic radicals, we chose several secondary aryl amines and tertiary amines as precursors that are known to produce radicals via chemical or electrochemical methods. In particular, we show that stable aminium and aminyl radicals can be selectively produced from the same secondary aryl amine precursor using different solvent compositions that create different electron and hole population imbalance conditions. For the photoexcitation with visible light that is not absorbed by solvent and amine precursors, all the reactions were carried out using CsPbBr_3_ NCs, exhibiting exciton transition in visible wavelengths, although CsPbCl_3_ NCs with a higher band gap can perform the same reactions. The absorption spectrum and a TEM image of CsPbBr_3_ NCs are in the Supporting Information (Figure S1). Reducing the size of CsPbBr_3_ NCs to impose quantum confinement may affect the kinetics of the charge transfer reaction by altering the band edge level and the available surface area on the NCs. However, the impact may not be as pronounced as in the photoreaction mediated by the Dexter-type energy transfer from CsPbX_3_ NCs to triplet acceptors in a recent study.^29^ We chose CH_2_Br_2_ for dihalomethane solvent since the self-halide exchange between CsPbBr_3_ NCs and Br^–^ allows us to avoid changing the band gap of the NCs that depends on the halide composition.^25^ The identities of all the reaction products were confirmed by using the combination of UV–visible absorption, electron paramagnetic resonance (EPR), and mass spectrometry (MS), as detailed in the Supporting Information. MS data provided particularly informative and detailed structural information on the radical species due to its high sensitivity and specificity for product ion analysis in a mixture.

Table 1 summarizes the structure of the precursors and radicals formed using CsPbBr_3_ NCs as the photocatalyst under weak 473 nm excitation with the corresponding solvent composition. A notable result summarized in Table 1 is that two different radical species are formed under different solvent conditions from the same secondary aryl amine precursor, such as phenothiazine (PTZ) and phenoxazine (POZ). When a 1:1 v/v mixture of hexane and CH_2_Br_2_ is used as the solvent, the aminium radical is formed exclusively via one-electron oxidation. Although CH_2_Br_2_ does the same reaction, 50% diluted CH_2_Br_2_ in hexane was used to improve the dispersion of the NCs in solvent. When only hexane is used as the solvent, the aminyl radical is formed instead of the aminium radical, which is the hydrogen atom abstraction product of the same precursor. In the CH_2_Br_2_/hexane solvent mixture, electrons in the photoexcited CsPbBr_3_ NCs were removed irreversibly, resulting in the effective one-electron oxidation of amines via hole transfer undisturbed by the electrons at the conduction band (Scheme 1a). The combination of the reductive dissociation of CH_2_Br_2_ and the photoinduced halide self-exchange is considered to create a kinetically favorable pathway that consumes electrons by forming the gaseous reduction products of CH_2_Br_2_ over the reversible back electron transfer to NCs. Earlier studies reported the observation of methane and ethylene produced as the electrochemical reduction of CH_2_Br_2._^30,31^ In contrast, when only hexane is used as the solvent, both electrons and holes are available for the oxidative and reductive charge transfer processes. The initial oxidation followed by the reductive deprotonation breaking the N–H bond is a plausible pathway to form an aminyl radical,^32,33^ which is in competition with the sequential oxidation and reduction recovering the initial precursor (Scheme 1b).

**Table 1:**
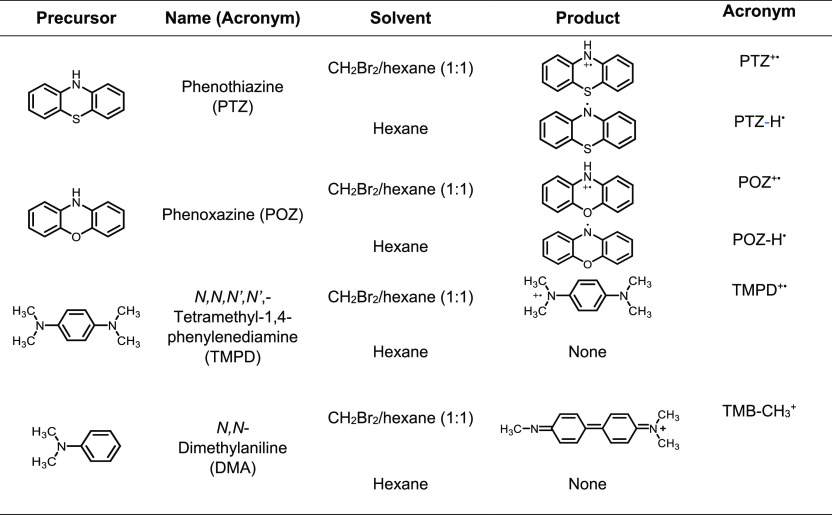
Structure of the Precursor Amines and the Radicals Formed by CsPbBr_3_ NCs Dispersed in the Given Solvent under 473 nm Excitation

**Figure 1:**
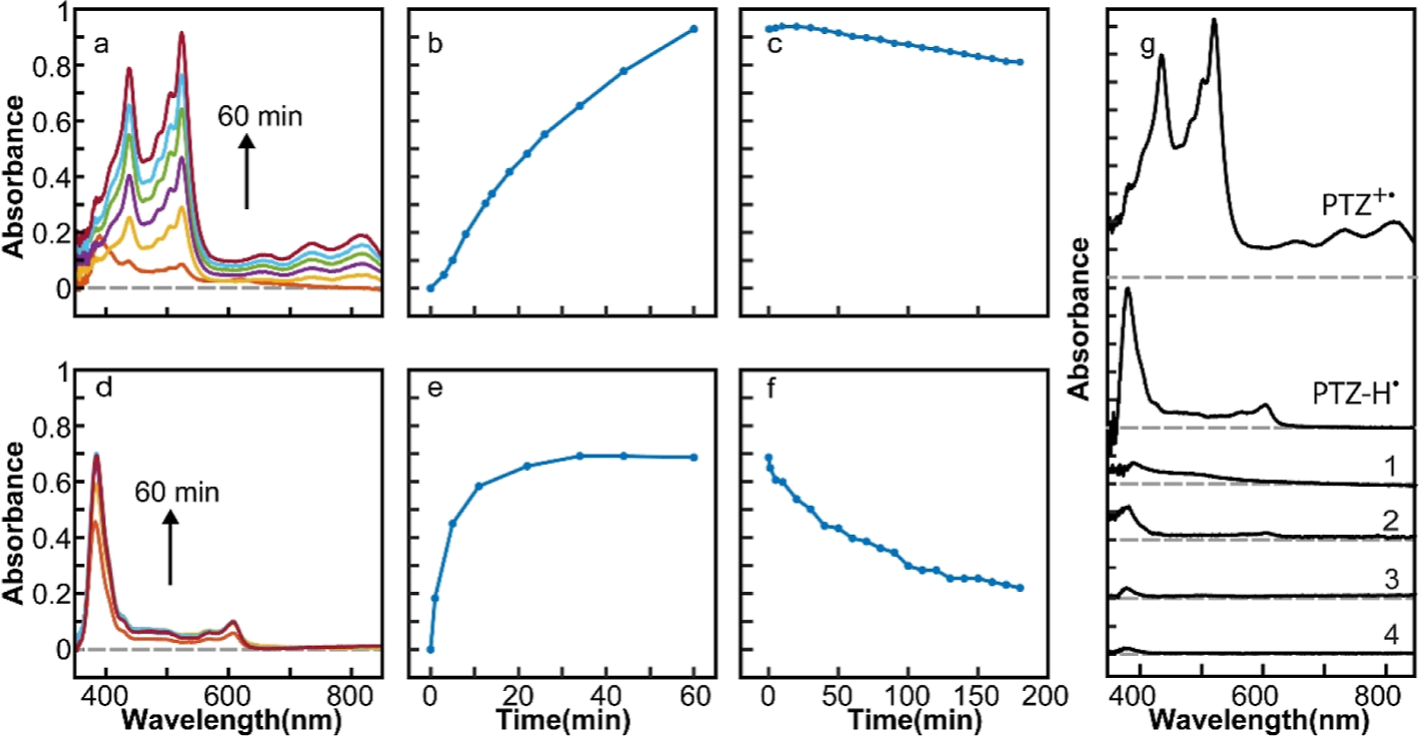
(a,d) Time-dependent absorption spectra of the photogenerated PTZ^+•^ (a) and PTZ-H^•^ (d). (b,e) Time-dependent absorption intensity of PTZ^+•^ at 523 nm (b) and PTZ-H^•^ at 387 nm (e). (c,f) Decay of the absorption intensity from PTZ^+•^ at 523 nm (c) and PTZ-H^•^ at 387 nm (f) in the dark. (g) Absorption spectra of the products after 1 h of reaction in four additional reaction conditions compared with those of PTZ^+•^ and PTZ-H^•^. The combinations of the QD photocatalyst and solvent for the additional reaction conditions are (1) CsPbBr_3_ + PTZ + BQ in hexane; (2) CdSSe + PTZ in hexane; (3) CdSSe + PTZ + BQ in hexane; and (4) CdSSe + PTZ in CH_2_Br_2_/hexane.

To examine the capability of CsPbBr_3_ NCs to produce the radicals stably under the reaction conditions listed in Table 1, the progress of the reaction was monitored by measuring the characteristic absorption of each radical species. The stability of the radicals was also examined by monitoring the dissipation of the absorption from the photogenerated radicals in the dark after the photoexcitation. In addition, ex situ EPR spectroscopy further confirmed the presence of the radical species that could coexist stably with NCs in the solution (Figure S2). Figure 1a,d shows the time-dependent absorption spectra of the reaction products from the mixture of PTZ and CsPbBr_3_ NCs in two different solvents, i.e., the CH_2_Br_2_/hexane mixture and hexane, under 473 nm excitation. For clarity, the absorption from CsPbBr_3_ NCs that did not change during the reaction was subtracted from all raw absorption spectra to show only the net changes in the absorption in Figure 1a,d. The raw absorption spectra at different reaction times are in the Supporting Information (Figure S3). CsPbBr_3_ NCs were stable without exhibiting any sign of degradation during the reaction (>3 h), likely due to the efficient removal of both charge carriers. Since PTZ does not exhibit a measurable absorption at wavelengths longer than 360 nm, the spectra shown in Figure 1a,d reflect only the reaction product of PTZ, which match well with the reported absorption spectra of aminium (PTZ^+•^) and aminyl (PTZ-H^•^) radicals, respectively.^34,35^

Figure 1b,e shows the time-dependent absorption intensities of the two radical species measured at 523 and 387 nm, respectively, corresponding to the peak of absorption from PTZ^+•^ and PTZ-H^•^. The increasing absorption intensity with the reaction time in Figure 1b indicates that PTZ^+•^ is continuously produced and accumulates in the solution. When the photoexcitation is discontinued, the absorption intensity of PTZ^+•^ decreases by only ∼10% after 3 h, as shown in Figure 1c, demonstrating the stability of PTZ^+•^. The time-dependent absorption of PTZ-H^•^ shown in Figure 1e exhibits a more rapid increase and saturation under the same photoexcitation condition. When the photoexcitation is discontinued, the absorption intensity of PTZ-H^•^ decays faster than that of PTZ^+^,^•^ diminishing by ∼65% after 3 h (Figure 1f) indicating that PTZ^+•^ is more stable than PTZ-H^•^ under the employed reaction condition. It is informative to compare the absorption spectra of PTZ^+•^ and PTZ-H^•^ with those obtained under several additional control reaction conditions. In Figure 1g, absorption spectra taken after 1 h of reaction under four additional reaction conditions are compared with the spectra of PTZ^+•^ and PTZ-H^•^ at 1 h of reaction time on the same absorbance scale. The details of the additional control reaction conditions are given in the caption of Figure 1g. One is using benzoquinone (BQ) as the electron acceptor in the hexane solution of CsPbBr_3_ NCs (condition 1), which exhibits only a weak and indistinct absorption after 1 h of reaction. While BQ accepts the electron from CsPbBr_3_ NCs, the electron can be transferred back from the reduced BQ (BQ^–•^) to the valence band of the NCs removing the hole or to other electron acceptors that may exist in the solvent medium. A recent study reported ∼65 ps electron transfer time from CsPbBr_3_ NCs to BQ and ∼50 ps hole transfer time from CsPbBr_3_ NCs to PTZ at the concentrations of BQ and PTZ comparable to this study (5 mM).^36^ The same study also reported a nanosecond time scale for the charge recombination between CsPbBr_3_ NCs and BQ^–•^ or PTZ^+•^. The fact that neither PTZ^+•^ nor PTZ-H^•^ is observable in the absorption spectrum of condition 1 suggests that the back electron transfer from BQ^–•^ to PTZ^+•^ may be the dominant pathway prohibiting the production of any radical species, which contrasts to the reaction in Scheme 1a with an irreversible electron removal pathway.

For conditions 2–4, CsPbBr_3_ NCs were replaced with CdSSe NCs having the same band gap and similar ligands as those of CsPbBr_3_ NCs under three different solvent compositions, i.e., hexane, hexane with BQ as the electron acceptor, and the CH_2_Br_2_/hexane mixture. The absorbance of CdSSe NCs at the excitation wavelength (473 nm) was matched to that of CsPbBr_3_ NCs to produce the same total number of photoexcited charge carriers by the NCs under all reaction conditions. Only in hexane solvent (condition 2), a weak absorption feature that can be attributed to PTZ-H^•^ is observed analogously to the case of CsPbBr_3_ NCs in hexane, albeit at a much lower intensity. The other two reaction conditions (condition 3 and 4) did not produce any significant product identifiable in the absorption spectra. CdS NCs with the valence bandedge level close to that of CsPbBr_3_ NCs^37^ were also ineffective in generating radicals in a separate measurement. The above comparison suggests that the ability to irreversibly remove electrons is the key to the difference between the two NCs in their ability to oxidatively generate aminium radicals, rather than the thermodynamic potential for charge transfer. In the earlier studies, the reduction of the dissolved O_2_ in solvent was proposed to be capable of removing the photoexcited electrons from CsPbBr_3_ NCs driving the oxidative reaction.^16,17^ However, in the present study, which generates stable radicals from amines, O_2_ does not play a significant role in consuming the photoexcited electrons necessary to perform the oxidative aminium radical formation. In the absence of CH_2_Br_2_ in the solvent, CsPbBr_3_ NCs do not produce PTZ^+•^ but produce PTZ-H^•^ even in the presence of dissolved O_2_, while the presence of O_2_ adds more complexity in the reaction (Figure S4).

**Figure 2:**
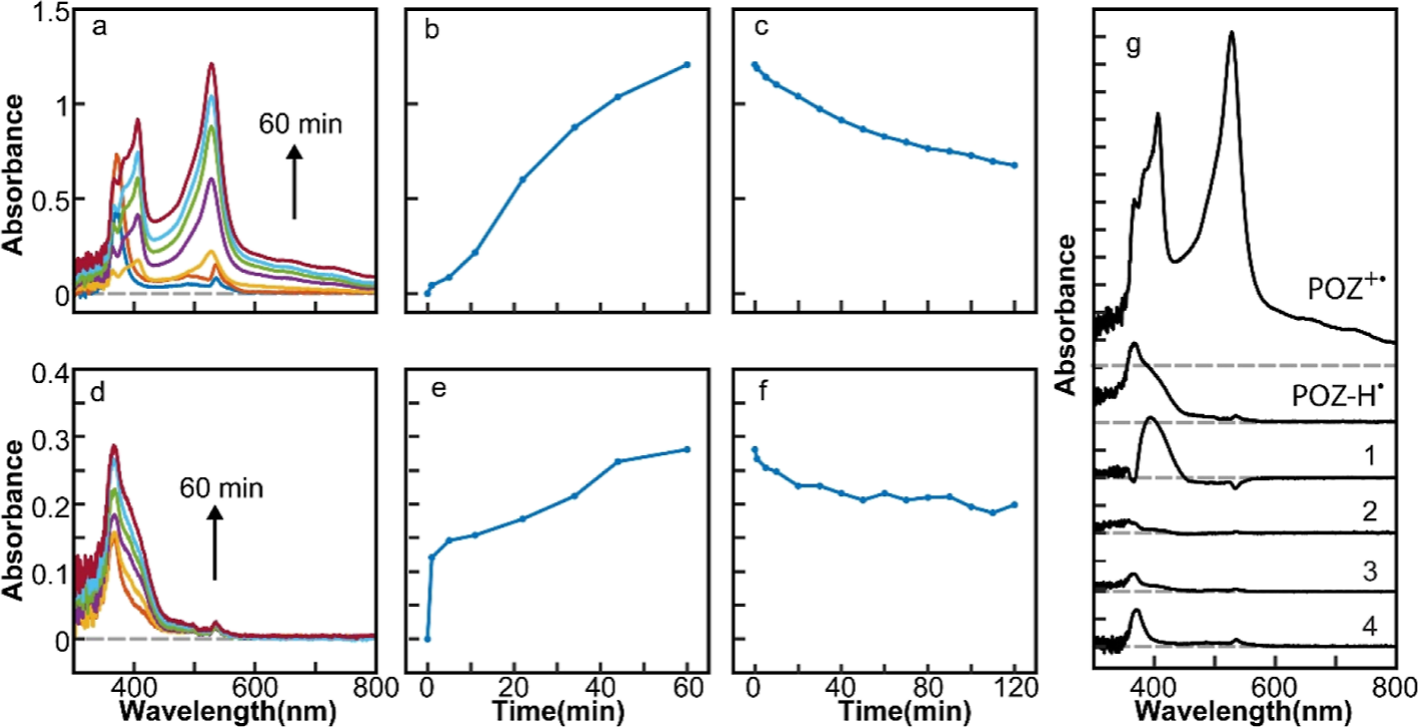
(a,d) Time-dependent absorption spectra of the photogenerated POZ^+•^ (a) and POZ-H^•^ (d). (b,e) Time-dependent absorption intensity of POZ^+•^ at 528 nm (b) and POZ-H^•^ at 367 nm (e). (c,f) Decay of the absorption from POZ^+•^ at 528 nm (c) and POZ-H^•^ at 367 nm (f) in the dark. (g) Absorption spectra of the products after 1 h of reaction in four additional reaction conditions compared with those of POZ^+•^ and POZ-H^•^. The combinations of the QD photocatalyst and solvent for the additional reaction conditions are (1) CsPbBr_3_ + POZ + BQ in hexane; (2) CdSSe + POZ in hexane; (3) CdSSe + POZ + BQ in hexane; and (4) CdSSe + POZ in CH_2_Br_2_/hexane.

The selective production of aminium and aminyl radicals by CsPbBr_3_ NCs by introducing charge carrier population imbalance was also possible for POZ, another secondary aryl amine. Figure 2a,d shows the time-dependent absorption spectra of the reaction products from the mixture of POZ and CsPbBr_3_ NCs in two different solvents under the same photoexcitation condition as in PTZ. The absorption peaks (380, 407, and 528 nm) in Figure 2a are characteristic of POZ^+•^.^38,39^ The peak (367 nm) in Figure 2d is attributed to POZ-H^•^.^39,40^Figure 2b,e shows the time-dependent absorption intensities of the two radical species measured at 528 and 367 nm from POZ^+•^ and POZ-H^•^, respectively. Figure 2c,f shows the decay of absorption from POZ^+•^ and POZ-H^•^ in the dark over 2 h. While the time scales of the buildup and decay of the absorption intensities are different from those of PTZ, both PTZ and POZ show the same selective formation of different radicals in different solvents. In Figure 2g, absorption spectra obtained under four additional reaction conditions are shown with the spectra of POZ^+•^ and POZ-H^•^, similarly to the comparison made in Figure 1g. Adding BQ to the hexane solution of CsPbBr_3_ NCs (condition 1) results in a spectrum similar to that of POZ-H^•^, suggesting that the reductive deprotonation of POZ^+•^ by BQ^–•^ is more facile than that in the case of PTZ^+•^. As in the case of PTZ, CdSSe NCs are not effective in generating radical species under all solvent conditions (condition 2–4).

Halide exchange in CsPbBr_3_ NCs with Br^–^ generated from the reductive dissociation of CH_2_Br_2_ appears to be crucial for the irreversible removal of the electrons from CsPbBr_3_ NCs necessary for the stable accumulation of the photogenerated aminium radical. Our recent study showed that the photoexcited II–VI NCs also produce Br^–^ via the reductive dissociation of CH_2_Br_2_ similar to perovskite NCs.^25^ This was confirmed by observing the halide exchange in CsPbCl_3_ from the mixture of CsPbCl_3_ and CdSSe NCs in CH_2_Br_2_/hexane solvent, where 455 nm light excited CdSSe NCs but not CsPbCl_3_ NCs. The halide exchange in this NC mixture was much slower than in the directly photoexcited CsPbCl_3_ NCs in the same solvent since the halide ions produced by CdSSe NCs need to diffuse to the distant CsPbCl_3_ NCs. The inability of CdSSe NCs to generate PTZ^+•^ indicates that the electron transfer to CH_2_Br_2_ and subsequent generation of CH_2_Br^•^ and Br^–^ are insufficient for the irreversible removal of electrons from the NCs, presumably due to the dominant geminate recombination of CH_2_Br^•^ and Br^–^ and back electron transfer to the NCs. Therefore, we conclude that the halide exchange by CsPbBr_3_ NCs is necessary to enable the irreversible removal of electrons from CsPbBr_3_ NCs. We postulate that the role of halide exchange by CsPbBr_3_ NCs is the immediate takeup of Br^–^ generated from the reductive dissociation of CH_2_Br_2_ and delayed release away from the site of generation, thus suppressing the geminate recombination of CH_2_Br^•^ and Br^–^. This would favor the irreversible removal of electrons from CsPbBr_3_ NCs, enabling the stable accumulation of the photogenerated aminium radical. When the mixture of CsPbCl_3_ and CdSSe NCs in CH_2_Br_2_/hexane solvent is used under 455 nm excitation, only PTZ-H^•^ was formed, similarly to CdSSe NCs alone (Figure S5). This indicates that the efficient exchange of Br^–^ at the site of its generation is needed to drive the one-electron oxidation of PTZ by irreversibly removing the electrons.

The generation of the aminyl radical (PTZ-H^•^) requires the transfer of both charge carriers with the net result of N–H bond breaking. It is intriguing that CsPbBr_3_ NCs are significantly more effective than II–VI NCs since both NCs can transfer electrons and holes. The kinetics of PTZ-H^•^ formation depend on the competition of the multiple interfacial processes and the diffusion of the reactant to the NC surface. The charge transfer rate in the NC-PTZ (PTZ^+•^) pair, the bond dissociation rate in the NC-PTZ^+•^ pair, and the effective concentration of the reactant at the NC surface, determined by the NC’s electronic structure and the surface-bound ligand, all contribute to the PTZ-H^•^ production rate. Therefore, it is difficult to identify all the factors responsible for the difference observed between the two different NCs. Nevertheless, a comparative study described below suggests that the charge of the surface-terminating ions of the NC lattice may play a significant role in the reductive N–H bond breaking of PTZ^+•^ to form PTZ-H^•^.

**Figure 3:**
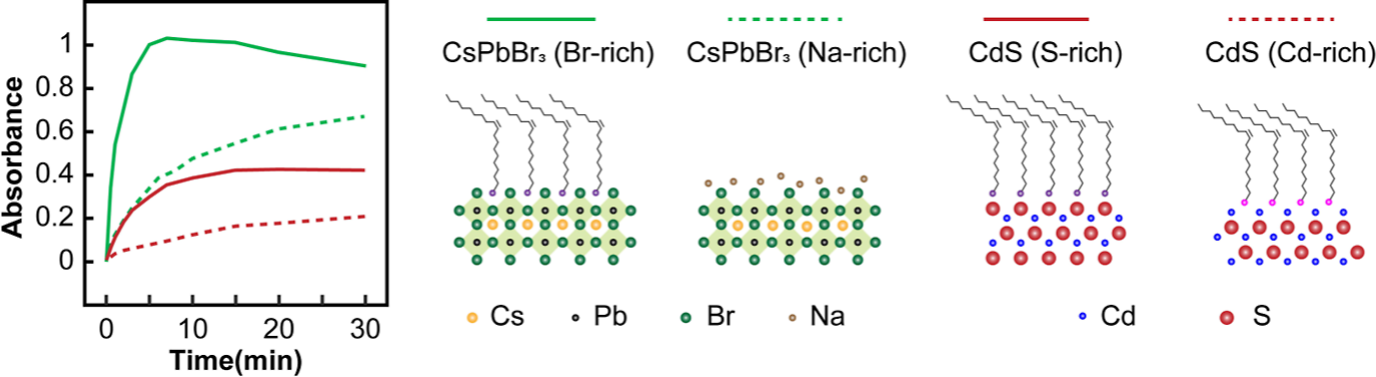
Time-dependent absorbance of PTZ-H^•^ at 387 nm produced by CsPbBr_3_ (green) and CdS (red) NCs with an anion-rich (solid) and cation-rich (dashed) lattice surface. For this comparison, all four NCs were excited at the same excitation rate. The illustration of the NC surface is for the OLAB-passivated CsPbBr_3_ NCs with a Br^–^-rich surface, the CsPbBr_3_ NCs with a Na^+^-rich surface without organic ligands, the oleylamine-passivated CdS NCs with a S^2–^-rich surface, and the oleic-acid-passivated CdS NCs with a Cd^2+^-rich surface.

We hypothesize that the reductive N–H bond breaking of the intermediate species (PTZ^+•^) requires interaction with the NC surface acting as an active site since a simple one-electron reduction of PTZ^+•^ would recover the initial reactant (PTZ). We further hypothesize that the anion-terminated NC surface may provide a better environment for the positively charged PTZ^+•^ to interact with the NC surface. Since CsPbBr_3_ and CdS NCs prepared using typical synthesis methods have Br^–^-rich and Cd^2+^-rich surfaces, respectively, for the higher photoluminescence intensity, we examined the effect of having the ions of the opposite charge on the surface of each NC sample. To this end, we compared the initial production rate of PTZ-H^•^ by CsPbBr_3_ and CdS NCs each with both cation-rich and anion-rich lattice surfaces, as shown in Figure 3. The details of the difference in the properties of NCs with the cation-rich and anion-rich lattice surface are in the Supporting Information (Figures S6 and S7). While the role of the ligand headgroup cannot be easily separated, we found that Br^–^-terminated CsPbBr_3_ NCs passivated with oleylammonium bromide (OLAB) are more effective in producing PTZ-H^•^ than Na^+^-terminated CsPbBr_3_ NCs without long surface ligands, despite the more steric hindrance on CsPbBr_3_ NCs with the OLAB ligand. Similarly, CdS NCs produce PTZ-H^•^ more effectively when the NC surface is anion-rich for similar surface ligands (oleylamine vs oleic acid). Our results indicate that PTZ-H^•^ generation via multistep charge transfer and bond dissociation can be altered by varying the surface atoms of the NC, although further studies are needed to draw a more definitive conclusion. It is noteworthy that an earlier study observed the enhancement of diamine oxidation and cyclization by introducing Cu^2+^ ions to CsPbBr_3_ NCs that provided binding sites for the reactant.^17^ These suggest the possibility of further tuning the radical formation reactions by varying the surface ion composition of CsPbX_3_ NCs as well as the ligand that has shown the ability to exhibit selective reaction such as in stereoselective radical coupling.^16^

**Figure 4:**
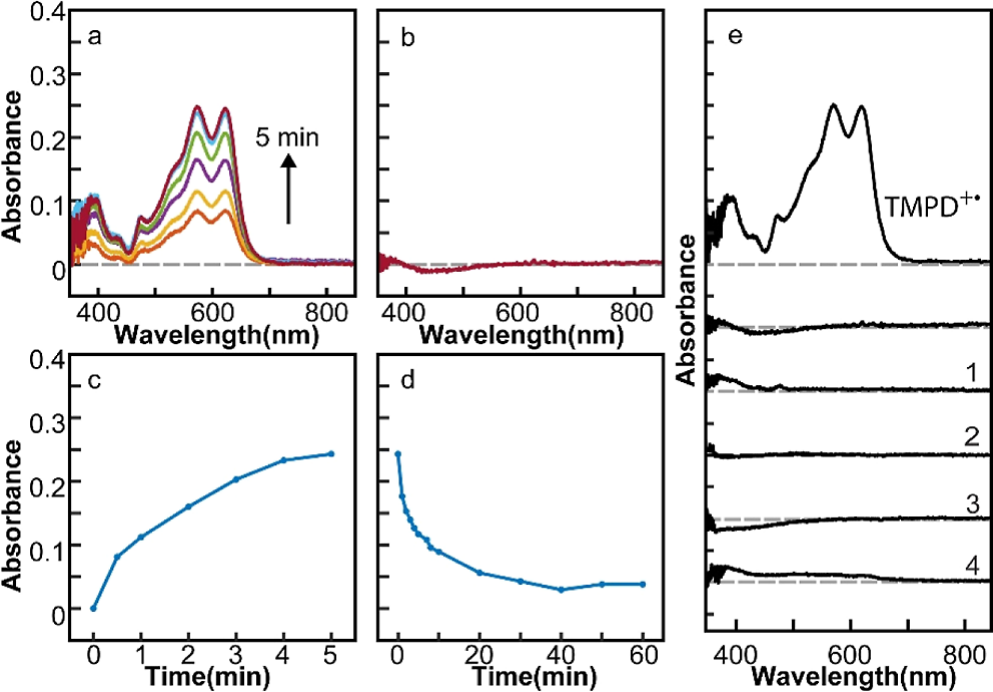
(a) Time-dependent absorption spectra of the photogenerated TMPD^+•^. (b) Absorption spectra of the product after 5 min of reaction for TMPD^+^ CsPbBr_3_ NCs in hexane. (c) Time-dependent absorption intensity of TMPD^+•^ at 624 nm. (d) Decay of the absorption from TMPD^+•^ at 624 nm in the dark. (e) Absorption spectra of the products after 5 min of reaction in four additional reaction conditions compared with that of TMPD^+•^. The combinations of the QD photocatalyst and solvent for the additional reaction conditions are (1) CsPbBr_3_ + TMPD + BQ in hexane; (2) CdSSe + TMPD in hexane; (3) CdSSe + TMPD + BQ in hexane; and (4) CdSSe + TMPD in CH_2_Br_2_/hexane.

For the tertiary amines, only the production of aminium radical via one-electron oxidation is possible because of the absence of the N–H bond necessary to produce aminyl radical. We examined the photoinduced reaction product of *N*,*N*,*N*′,*N*′-tetramethyl-1,4-phenylenediamine (TMPD) and *N*,*N*-dimethylaniline (DMA) to confirm the production of the radical species in the CH_2_Br_2_/hexane mixture. These two amines are chosen since they are known to produce aminium radicals electrochemically or chemically.^41–44^Figure 4a,b shows the time-dependent absorption spectra of the reaction products from the mixture of TMPD and CsPbBr_3_ NCs in two different solvents under 473 nm excitation. TMPD^+•^ was formed by CsPbBr_3_ NCs in the CH_2_Br_2_/hexane mixture via hole transfer without interference from electron transfer. The absorption features in Figure 4a at 575 and 624 nm are attributed to TMPD^+•^.^35,41^ In contrast, CsPbBr_3_ NCs in hexane do not form TMPD^+•^ or any other product identifiable from the absorption spectra. Even when BQ is used as the electron acceptor in a hexane solution of CsPbBr_3_ NCs, TMPD^+•^ was not formed similarly to the case of PTZ (Figure 4e, condition 1). Dication of TMPD, known to form on the electrode via two-electron oxidation, is not observed, likely due to the higher oxidation potential for the second hole transfer.^45^ When the photoexcitation is discontinued, the absorption from TMPD^+•^ decayed on the time scale of 10 min. When CdSSe NCs are used instead of CsPbBr_3_ NCs, the production of TMPD^+•^ was suppressed under all three solvent conditions (Figure 4e, conditions 2–4), consistent with the observations made for other amines discussed above.

The quantum yield of photogeneration of PTZ^+•^ and TMPD^+•^ is in a 5–10% range under our experimental conditions and comparable to that of the molecular catalyst.^46^ (Calculation in the Supporting Information) Since the interfacial charge transfer to the precursor molecules can be affected by the surface ligands, surface modification that gives the easier access of the precursor to the surface of the NCs should increase the quantum yield.^47^ While we plan to study the kinetics of radical formation correlated with the NC structure, ligand, and solvent polarity in the future, we confirmed that partially removing the long-chain ligand (oleylammonium bromide) can already increase the quantum yield from ∼5 to >30% for PTZ^+•^ (Figure S8).

**Figure 5:**
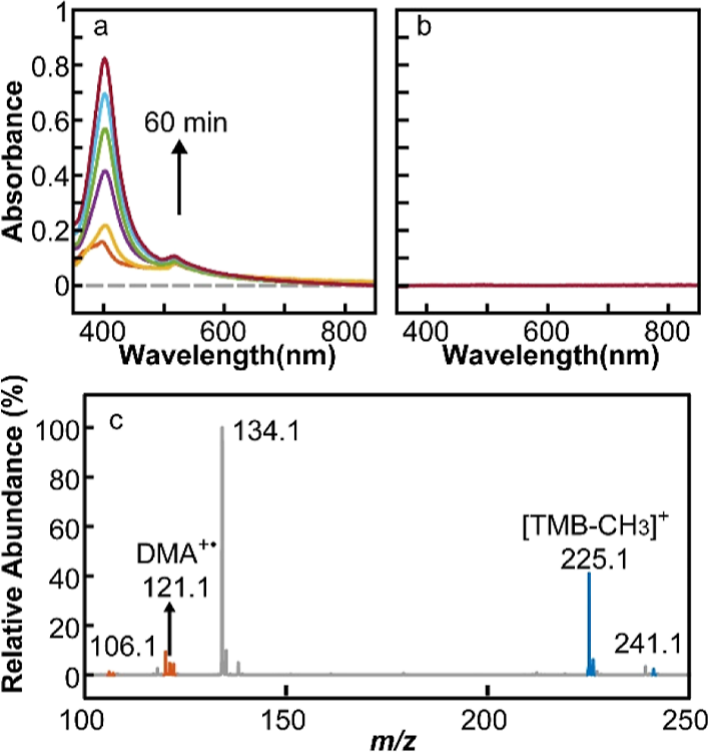
(a) Time-dependent absorption spectra of the reaction product of DMA in CH_2_Br_2_/hexane solution of CsPbBr_3_ NCs. (b) Absorption spectrum of the product of DMA in hexane solution of CsPbBr_3_ NCs or CdSSe QDs in CH_2_Br_2_/hexane after 1 h of reaction. (c) Mass spectrum of the reaction product of DMA in CH_2_Br_2_/hexane solution of CsPbBr_3_ NCs.

Figure 5a shows the time-dependent absorption spectra of the reaction product from a mixture of DMA and CsPbBr_3_ NCs in CH_2_Br_2_/hexane under 473 nm excitation. Unlike in the case of TMPD, the absorption spectra in Figure 5a do not correspond to DMA^+•^. The absence of DMA^+•^ is consistent with its short lifetime (∼μs) reported from the electrochemical oxidation studies of DMA.^48,49^ As will be discussed further below, the reaction product has been identified as the structure resulting from the dimerization of DMA^+•^ and additional demethylation. No product detectable from UV–visible absorption was formed when only hexane was used as the solvent (Figure 5b) or when CsPbBr_3_ NCs were replaced with CdSSe NCs.

**Scheme 2:**
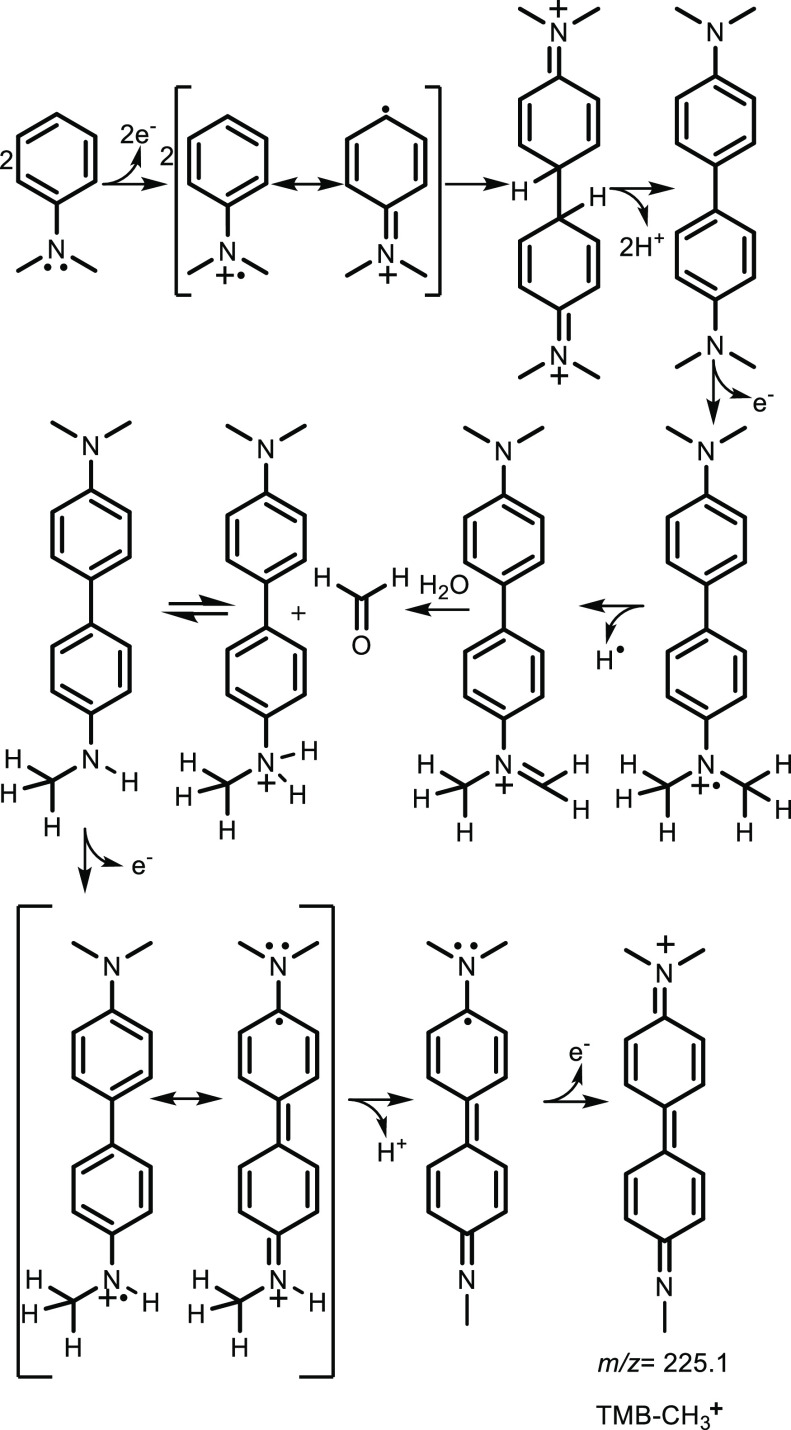
Proposed Pathway to Form TMB-CH_3_^+^

Because of the limited structural information that can be obtained from the absorption spectra in Figure 5a, the structure of the product was determined from mass spectrometry, as described in Supporting Information (Experimental Section). In typical electrochemical oxidation of DMA, the observed reaction products are *N*,*N*,*N*′,*N*′-tetramethylbenzidine (TMB) resulting from the dimerization of DMA^+•^ or its single- or two-electron oxidation product (TMB^+•^ or TMB^++^) depending on the electrode potential.^48^ In contrast, the photoinduced oxidation of DMA by CsPbBr_3_ NCs produces only the demethylation product of TMB (TMB-CH_3_^+^), where one of the amine groups transforms into imine. Scheme 2 shows the proposed pathway of producing TMB-CH_3_^+^, which is analogous to the oxidative *N*-dealkylation of amines catalyzed by enzymes and biomolecules.^50–52^ In the mass spectrum of the reaction product shown in Figure 5c, the species at *m*/*z* 225.1 correspond to TMB-CH_3_^+^. In contrast, the peak at *m*/*z* 240.1 corresponding to TMB^+•^ that signifies the formation of TMB is absent. The identification of the species at *m*/*z* 225.1 as TMB-CH_3_^+^ was made from the tandem mass spectrometry study by fragmenting the species at *m*/*z* 225.1 via collision-induced dissociation (CID) into two species whose structures have been determined from the mass spectra. Further discussions on the structural determination are in the Supporting Information (Figure S9).

**Figure 6:**
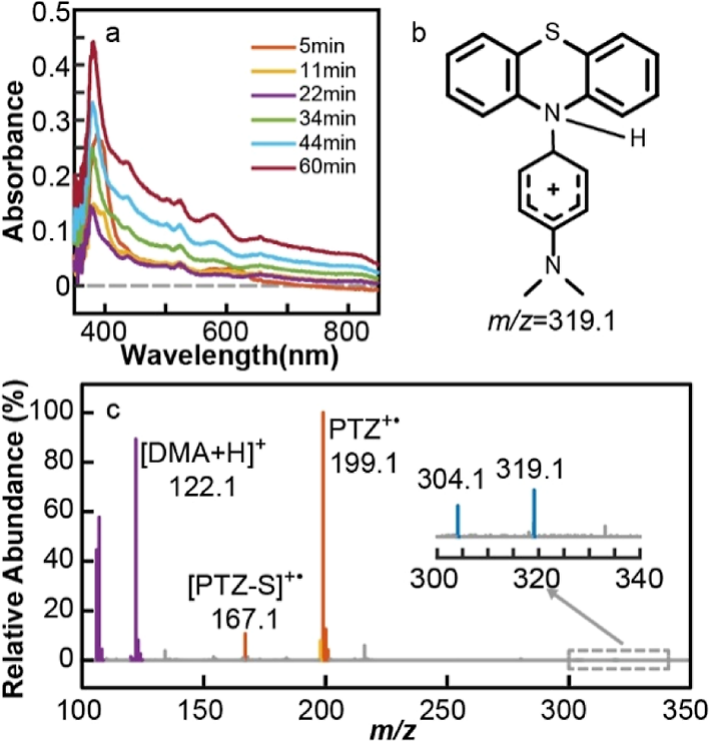
(a) Time-dependent absorption spectra of the reaction product of the mixture of DMA and PTZ in CH_2_Br_2_/hexane solution of CsPbBr_3_ NCs under 473 nm excitation. (b) Structure of the coupling product that corresponds to *m*/*z* 319.1 in the MS spectrum. (c) Mass spectrum of the reaction product from the mixture of DMA and PTZ in CH_2_Br_2_/hexane solution of CsPbBr_3_ NCs after 1 h of photoexcitation at 473 nm.

To further examine if the coupling reaction between two different radical species can be achieved using CsPbBr_3_ NCs, we examined the photoinduced reaction product from the mixture of DMA and PTZ in a solution of CsPbBr_3_ NCs. The combination of DMA and PTZ is chosen because it has been demonstrated in previous studies that the radicals of DMA and PTZ generated via electrochemical oxidation can undergo a coupling reaction.^42,53^ The C–N bond formation resulting from the coupling of the two radicals derived from DMA and PTZ also bears a practical importance for the synthesis of pharmaceuticals.^53^Figure 6a shows the time-dependent absorption spectra of the mixture of PTZ, DMA, and CsPbBr_3_ NCs in CH_2_Br_2_/hexane under 473 nm excitation, which is the reaction condition that can produce radicals from both PTZ and DMA. Since the identity of the coupling product cannot be determined from the absorption spectra, mass spectrometry was used to determine the structure of the coupling reaction product. The mass spectrum of the reaction products (Figure 6c) indicates that the composition of the products is significantly more complex than that of a single precursor, exhibiting the species derived from each precursor in addition to the coupling product. In the mass spectrum, the species at *m*/*z* 319.1 corresponds to the coupling product between the radicals derived from PTZ and DMA, which was previously seen from the electrochemical coupling of DMA^+•^ and PTZ-H^•^.^42^ The absence of the peaks at *m*/*z* 240.1 (TMB^+•^) and *m*/*z* 225.1 (TMB-CH_3_^+^) in the mass spectrum indicates that the dimerization of DMA is suppressed when both DMA and PTZ are present in the reactant mixture. Since the reaction was performed in a fully oxidative condition in the CH_2_Br_2_/hexane mixture that limits the generation of PTZ-H^•^ proposed to be involved in the coupling reaction, the yield of the product is not optimal for this coupling reaction. Tuning the population of the specific radical species involved in the coupling reaction by varying the solvent composition and altering the degree of redox imbalance will be an interesting direction to explore further.

## Conclusions

We report a CsPbBr_3_ NC-based photocatalytic approach that can generate stable N-centered organic radicals from amines either oxidatively or via sequential oxidation and reductive N–H bond breaking in a selective manner without the interference from reversible charge transfer. This has been achieved by utilizing the unique capability of CsPbBr_3_ NCs to control the population imbalance of electrons and holes via photoinduced halide exchange between the NCs and CH_2_Br_2_ that facilitates irreversible electron removal from the photoexcited NCs. The ability to tune charge carrier population imbalance enabled the selective generation of aminium and aminyl radicals from the same secondary aryl amine precursor by varying only the solvent composition, which cannot be readily achieved with other semiconductor NC photocatalysts. In addition, coupling reactions between different N-centered radicals are also demonstrated by using CsPbBr_3_ NCs as the effective radical-generating photocatalyst.

## Methods

### Synthesis of OLAB-Passivated CsPbBr_3_ and Oleic Acid-Passivated CdSSe NCs

CsPbBr_3_ NCs (∼10 nm in size) were synthesized via hot injection following the previously reported method.^54^ Cs precursor solution (Cs-oleate solution) was prepared by heating the mixture of Cs_2_CO_3_ (250 mg), oleic acid (OA, 0.8 g), and 1-octadecene (ODE, 7 g) at 150 °C for >10 min under N_2_ flow on a Schlenk line. In a separate flask, Pb/Br precursor solution was prepared by dissolving PbBr_2_ (69 mg) in the mixture of oleylamine (OAm, 0.5 mL), OA (0.5 mL), and ODE (5 mL). Pb/Br precursor solution was heated at 120 °C for 10 min under vacuum in a Schlenk line, and the temperature was increased to 200 °C. Subsequently, 0.4 mL of Cs-oleate was injected into this solution to initiate the reaction. After a few seconds of reaction, the reaction was quenched with an ice water bath. The product was recovered by centrifuging the reaction mixture and redispersing the precipitate in hexane. The resulting NCs are Br^–^-rich on the surface.

CdSSe NCs were synthesized employing a previously reported method.^4,55^ S/Se precursor solution was prepared as S/Se = 10:1 in ODE. A 2 mL portion of the S/Se precursor solution was injected into the mixture of CdO (126 mg), ODE (12 mL), and OA (2.02 g) at 270 °C under N_2_ flow to initiate the reaction. After 4 min of reaction at 250 °C, the reaction was quenched by air cooling. The product was recovered by precipitating it with acetone and centrifugation. The precipitate was redispersed in toluene, followed by additional purification, with methanol as the antisolvent. The product is S^2–^-rich on the surface and passivated with oleic acid.

### Synthesis of CsPbBr_3_ NCs with a Na^+^-Rich Surface and CdS NCs with a S^2–^-Rich Surface

CsPbBr_3_ NCs with a Na^+^-rich surface were prepared by ligand exchange on the OLAB-passivated CsPbBr_3_ NCs following the method reported by Dong et al., which forms a stable bipolar shell, with Na^+^ occupying the outermost sites confirmed by the large increase of the positive ζ-potential.^56^ First, OLAB-passivated CsPbBr_3_ NCs were precipitated by adding methyl acetate and redispersed in toluene after centrifugation. This procedure was repeated three times to remove OLAB. Subsequently, 10 μL of 1 M phenylethylammonium bromide (PEAB) DMF solution was added and mixed vigorously. PEAB served as an intermediate ligand used before completely replacing the original ligand with NaBr. Finally, 10 μL of saturated NaBr DMF solution was added and mixed vigorously. After removing the excess salt by centrifugation, the product NCs were recovered by precipitation with methyl acetate and redispersed in toluene. No additional organic ligands replacing the OLAB are added, which drastically reduces the facet-to-facet distance of the NCs in the TEM image, as shown in Figure S7. CdS NCs with an S^2–^-rich surface were prepared by adding 10 μL of 1 M Na_2_S DMF solution and 4 μL of oleylamine to oleic acid-passivated CdS NCs that are Cd^2+^-rich on the surface. After removing the excess salt by centrifugation, the product NCs were recovered by precipitation with methyl acetate and redispersed in toluene.

### Photogeneration of Aminium and Aminyl Radicals by CsPbBr_3_ NCs

The stock solutions of PTZ, POZ, TMPD, DMA, and BQ were prepared by dispersing each compound in toluene at 50 mM concentration in a glovebox. All solvents (hexane or CH_2_Br_2_/hexane mixture) used to prepare the reactant mixture were bubbled with N_2_ for 30 min to remove the dissolved O_2_. For the photogeneration of the aminium radical, ∼1 mL of the reactant mixture containing CsPbBr_3_ NCs (absorbance of 0.1 at the excitation wavelength) and reactant amine (5 mM) dispersed in a CH_2_Br_2_/hexane mixture (1:1 v/v) was prepared in a sealed quartz cuvette. For the photogeneration of the aminyl radical, the reactant mixture was prepared in hexane instead of a CH_2_Br_2_/hexane mixture, while keeping the amount of other components identical. For the coupling reaction of DMA, ∼1 mL of the reactant mixture containing CsPbBr_3_ NCs (absorbance of 0.1 at excitation wavelength) and DMA (5 mM) was prepared in a sealed quartz cuvette with CH_2_Br_2_/hexane (volume ratio 1:1) as the solvent. For the coupling reaction of DMA with PTZ, ∼1 mL of the reactant mixture containing CsPbBr_3_ NCs (absorbance of 0.1 at excitation wavelength), DMA (5 mM), and PTZ (5 mM) dispersed in the CH_2_Br_2_/hexane solvent (volume ratio 1:1) was prepared in a sealed quartz cuvette. All of the reactant mixtures were purged with N_2_ for several minutes to further remove dissolved O_2_ before photoexcitation at 473 nm at the intensity of ∼1.7 mW/cm^2^. The concentration of BQ used in the control experiment was 5 mM. The absorbance of CdSSe NCs in the control experiments was also 0.1 at the excitation wavelength.

### Optical Measurements and Product Characterization

The absorption spectra of all of the solutions were measured with a CCD spectrometer (Ocean Optics, QE65). EPR spectra were obtained on a Bruker E1 Exsys with a super CW EPR bridge. EPR spectra of the radical products were obtained at room temperature at a 9.37 MHz microwave frequency and 20 mW microwave power. All mass spectrometry (MS) data were acquired on an Orbitrap Velos Pro mass spectrometer (Thermo Fisher Scientific). Raw data were extracted using the Qual Browser of the Xcalibur program (Thermo Fisher Scientific). The following MS parameters were used for all data acquisitions. Samples were analyzed through applications of 1.0–3 kV DC spray voltages. An S-lens RF level was set to be 67.9% for positive ion mode. The capillary temperature was set to be 280 °C. Full MS scans were acquired at a *m*/*z* range of 50–500. The resolution was set at 30,000. The microscan number was set at 2, and the maximum injection time was set at 200 ms. Tandem mass spectra were obtained via CID with a normalized collision energy ranging from 20 to 50 arbitrary units. All samples were analyzed via inductive nanoelectrospray ionization, where the electrode does contact the solution being analyzed. The electrode/solution contact was removed to ensure that the reaction of products observed via MS was completed by the photo-oxidation reaction and not from the transfer of electrons to/from the electrode. To fabricate the MS emitters used for analysis, World Precision Instrument borosilicate glass capillaries from Fisher Scientific were used and pulled to a fine tip using a micropipette puller (P-1000, Sutter Instruments). The following parameters were used to pull a capillary with an orifice size of >10 μm: heat = 530, pull = 0, velocity = 22, time = 250, pressure = 250, and ramp = 550.

## Supporting Information Available

The following information is available free of charge: The Supporting Information is available free of charge at https://pubs.acs.org/doi/10.1021/jacs.3c05323.

Chemical materials, radical generation QY calculation, additional absorption spectra, and additional MS spectra (PDF)
