# Supplementary material for: Efficient and Selective Photogeneration of Stable N-Centered Radicals via Controllable Charge Carrier Imbalance in Cesium Lead Halide Nanocrystals
Source: J Am Chem Soc. 2023 Jul 20;145(30):16862–71. doi: 10.1021/jacs.3c05323 (PMC10863071; doi:10.1021/jacs.3c05323)
Supplement: Supplementary file 1 — ja3c05323_si_001.pdf [file ja3c05323_si_001.pdf]

Supporting Information for

Efficient and Selective Photogeneration of Stable *N*-Centered Radicals via Controllable Charge Carrier Imbalance in Cesium Lead Halide Nanocrystals

Tian Qiao[1], Madison E. Edwards[1], Xueting Tang[1], Xin Yan[1] and Dong Hee Son[1,2]*

[1]Department of Chemistry, Texas A&M University, College Station, Texas, 77843, USA

[2]Center for Nanomedicine, Institute for Basic Science and Graduate Program of Nano Biomedical Engineering, Yonsei University, Seoul 03722, Republic of Korea

[*]Email: dhson@chem.tamu.edu

1. Chemicals

Cesium carbonate (Cs_2CO_3, puratronic, 99.994%, metals basis, Alfa Aesar), Lead (II) bromide ($PbBr_2$, puratronic, 99.999% metals basis, Alfa Aesar), Zinc bromide ($ZnBr_2$, 99.9%, metals basis, Alfa Aesar), Cadmium oxide (CdO, puratronic 99.998% trace metals basis, Alfa Aesar), Sulfur (99.98%, Sigma-Aldich), Selenium (200 mesh 99.999% trace metals basis, Alfa Aesar), Oleylamine (OAm, technical grade 70%, Sigma-Aldrich), Oleic acid (OA, technical grade 90%, Sigma-Aldrich), 1-Octadecene (ODE, technical grade 90%, Sigma-Aldrich), Tributyl phosphine (TBP, 95%, Alfa Aesar), Acetone (Certified ACS, Fischer), Methanol (99.5%, Millipore), Hexane (HPLC grade, Millipore), Dibromomethane (CH_2Br_2, 99%, Acros Organic), Toluene (anhydrous, 99.8%, Sigma-Aldrich), Phenothiazine (PTZ, 98+%, Alfa Aesar), Phenoxazine (POZ, 97%, Sigma-Aldrich), N,N,N′,N′-Tetramethyl-1,4-phenylenediamine (TMPD, 99%, Sigma-Aldrich), N,N-Dimethylaniline (DMA, 99%, Sigma-Aldrich), p-benzoquinone (BQ, 98+%, Alfa Aesar). All compounds were used as purchased without further purification.

2. Calculation of the quantum efficiency of generating radicals by the 473 nm laser with $CsPbBr_3$ NCs as the catalyst.

a. PTZ radical cation

After 10 mins of reaction, the absorbance of PTZ radical cation at 523 nm (A_{RC}) reaches 0.24. From the molar absorption coefficient (ε_{RC}) reported in the previous study,S[1] the number of PTZ radical cations (N_{RC}) is obtained from Beer's law:

$$A_{RC} = \varepsilon_{RC} C_{RC} b$$

$$0.24 = 6309.57\ M^{-1}cm^{-1} \bullet C_{RC} \bullet 1cm$$

$$C_{RC} = 3.80 \times 10^{-5} M$$

$$N_{RC} = C_{RC} \bullet 1mL \bullet 6.02 \times 10^{23} = 2.29 \times 10^{16}$$

The number of excitation photons (λ=473 nm, 1.7 mW incident intensity) absorbed by $CsPbBr_3$ NCs of the absorbance 0.1 at the excitation wavelength for 10 mins (N_{abs}) is calculated as follows. h is the Planck's constant and c is the speed of light.

$$N_{abs} = \frac{1.7\ mW \times 600s}{\left(\frac{hc}{\lambda}\right)} (1 - 10^{-0.1}) = 5.0 \times 10^{17}$$

Therefore, the quantum efficiency is:

$$\Phi = \frac{N_{RC}}{N_{abs}} = \frac{2.29 \times 10^{16}}{5.0 \times 10^{17}} = 4.6\%$$

b. TMPD radical cation

After 0.5 mins of reaction, the absorbance of TMPD radical cation at 624 nm (A_{RC}) reaches 0.081. From the molar absorption coefficient (ε_{RC}) reported in the previous study,[1] the number of TMPD radical cations (N_{RC}) is calculated using Beer's law:

$$A_{RC} = \varepsilon_{RC} C_{RC} b$$

$$0.081 = 12589.25 M^{-1} cm^{-1} \bullet C_{RC} \bullet 1cm$$

$$C_{RC} = 6.43 \times 10^{-6} M$$

$$N_{RC} = C_{RC} \bullet 1mL \bullet 6.02 \times 10^{23} = 3.87 \times 10^{15}$$

The number of excitation photons (λ=473 nm, 1.7 mW incident intensity) absorbed by $CsPbBr_3$ NCs of the absorbance 0.18 at the excitation wavelength for 0.5 mins (N_{abs}) is calculated as follows.

$$N_{abs} = \frac{1.7mW \times 30s}{\left(\frac{hc}{\lambda}\right)} (1 - 10^{-0.18}) = 4.12 \times 10^{16}$$

Therefore, the quantum efficiency is:

$$\Phi = \frac{N_{RC}}{N_{abs}} = \frac{3.87 \times 10^{15}}{4.12 \times 10^{16}} = 9.4\ \%$$

4. Additional figures

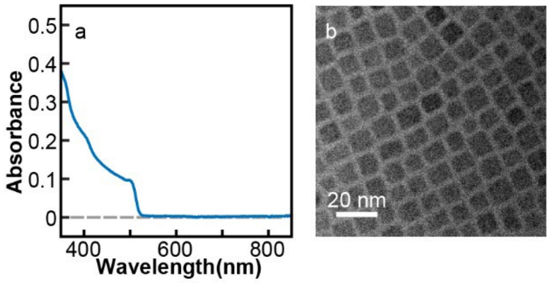

Figure S1. (a) Absorption spectrum of $CsPbBr_3$ NCs. (b) TEM image of $CsPbBr_3$ NCs.

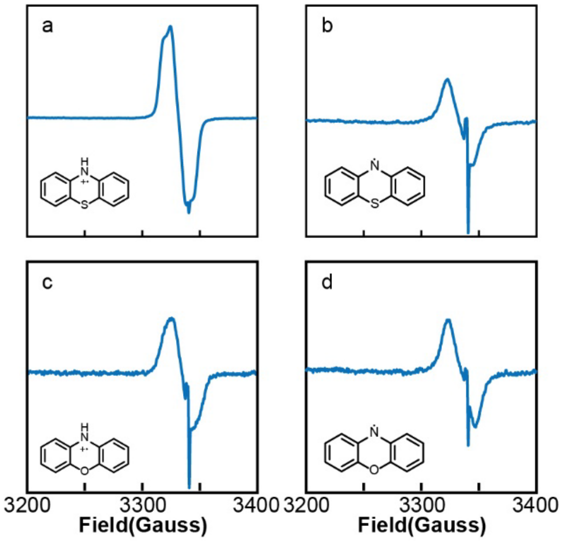

Figure S2. EPR spectrum of (a) $PTZ^{+\bullet}$, (b) PTZ-$H^{\bullet}$, (c) $POZ^{+\bullet}$, and (d) POZ-$H^{\bullet}$ generated by $CsPbBr_3$ NCs under 473 nm photoexcitation.

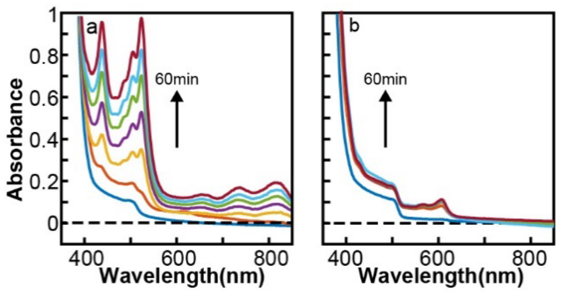

Figure S3. Raw time-dependent absorption spectra of (a) PTZ and $CsPbBr_3$ NCs in DBM/hexane and (b) PTZ and $CsPbBr_3$ in hexane that includes the absorption from $CsPbBr_3$ NCs.

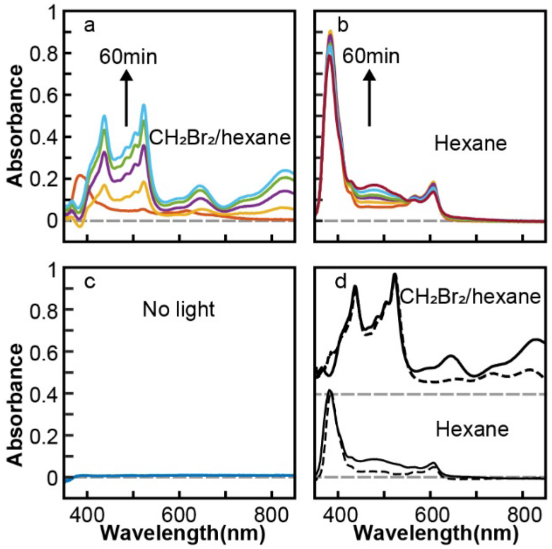

Figure S4. Time-dependent absorption spectra of (a) PTZ and $CsPbBr_3$ in DBM/hexane and (b) PTZ and $CsPbBr_3$ in hexane with the presence of oxygen in the reactant mixture. (c) Absorption spectrum shows that no oxidation product of PTZ was generated after 1 hour of reaction in dark in CH_2Br_2/hexane with the presence of oxygen. (d) Comparison of the oxidation product of PTZ with (solid) and without (dashed) the presence of oxygen in the reactant mixture after 1 hour of reaction. The absorption spectra of the reactions shown in the top half panel were carried out in CH_2Br_2/hexane and the ones in the bottom half panel were carried out in hexane. In both conditions, additional products with unknown identities were generated with the presence of oxygen.

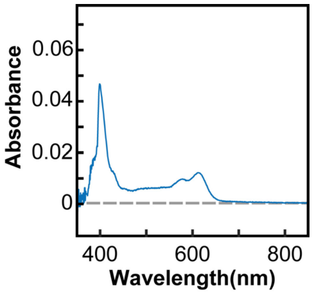

Figure S5. Absorption spectrum of the reaction product of PTZ using the mixture of $CsPbBr_3$ and CdSSe NCs excited at 455 nm (1.6 mW/cm^2, absorbance of CdSSe at 455 nm is 0.1) after 30 min of reaction. Only a small amount of PTZ-$H^{\bullet}$ is formed similarly to the case of CdSSe shown in Fig. 1g.

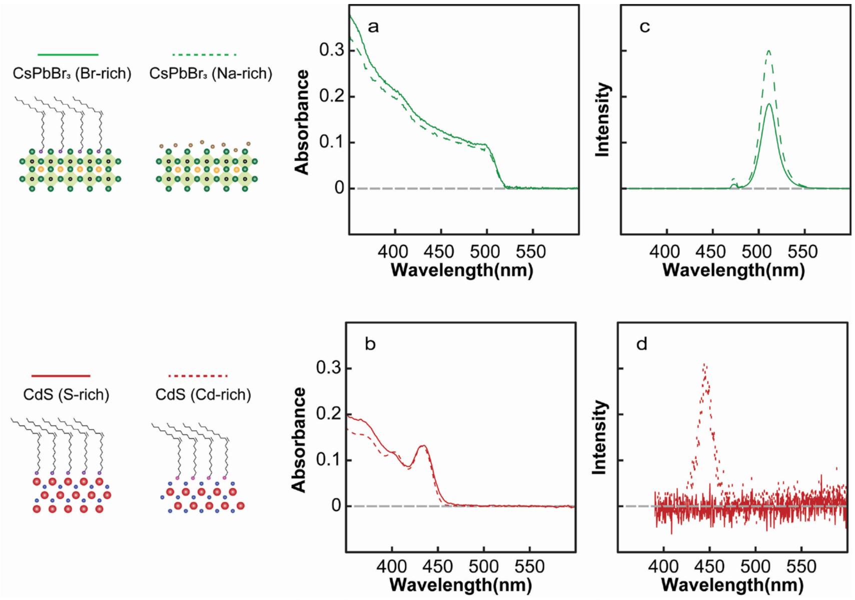

Figure S6. Absorption and PL spectra of (a, c) $CsPbBr_3$ NCs and (b, d) CdS NCs with different surface-terminating ions on the surface. Comparisons are made for OLAB-passivated $CsPbBr_3$ NCs with Br^-–rich surface and $CsPbBr_3$ NCs with Na^+–rich surface without organic ligands, oleylamine-passivated CdS NCs with S^{2-}–rich surface, and oleic acid-passivated CdS NCs with Cd^{2+}–rich surface. Oleylamine-passivated CdS NCs with S^{2-}–rich surface shows quenched PL since excess surface sulfur is known to trap holes.

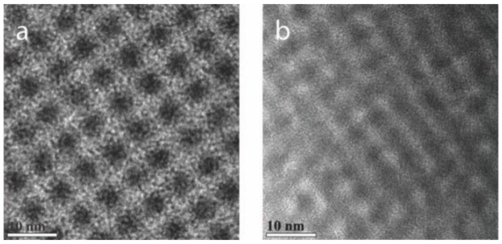

Figure S7. Comparison of the TEM images of OLAB-passivated 6nm $CsPbBr_3$ QDs with Br^-–rich surface (a) and 6 nm $CsPbBr_3$ QDs with Na^+–rich surface without organic ligands. Due to the absence of the organic ligand on the NCs shown in (b), facet-to-facet distance is reduced to ~0.5 nm from ~3 nm on TEM images consistent with the removal of long-chain organic ligands. For this comparison, smaller QDs that exhibit higher size uniformity are used for more facile comparison of the difference resulting from the removal of long-chain ligand.

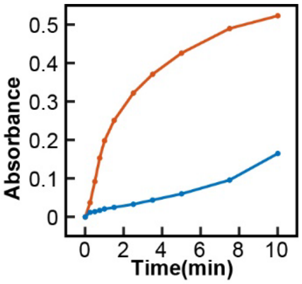

Figure S8. Comparison of the time-dependent absorbance of $PTZ^{+\bullet}$ photogenerated with $CsPbBr_3$ NCs under 473 nm excitation before (blue) and after (red) partially removing the surface ligands (OLAB) by rinsing the NCs with methyl acetate. The difference in the initial slope of the absorbance vs time is ~6 fold.

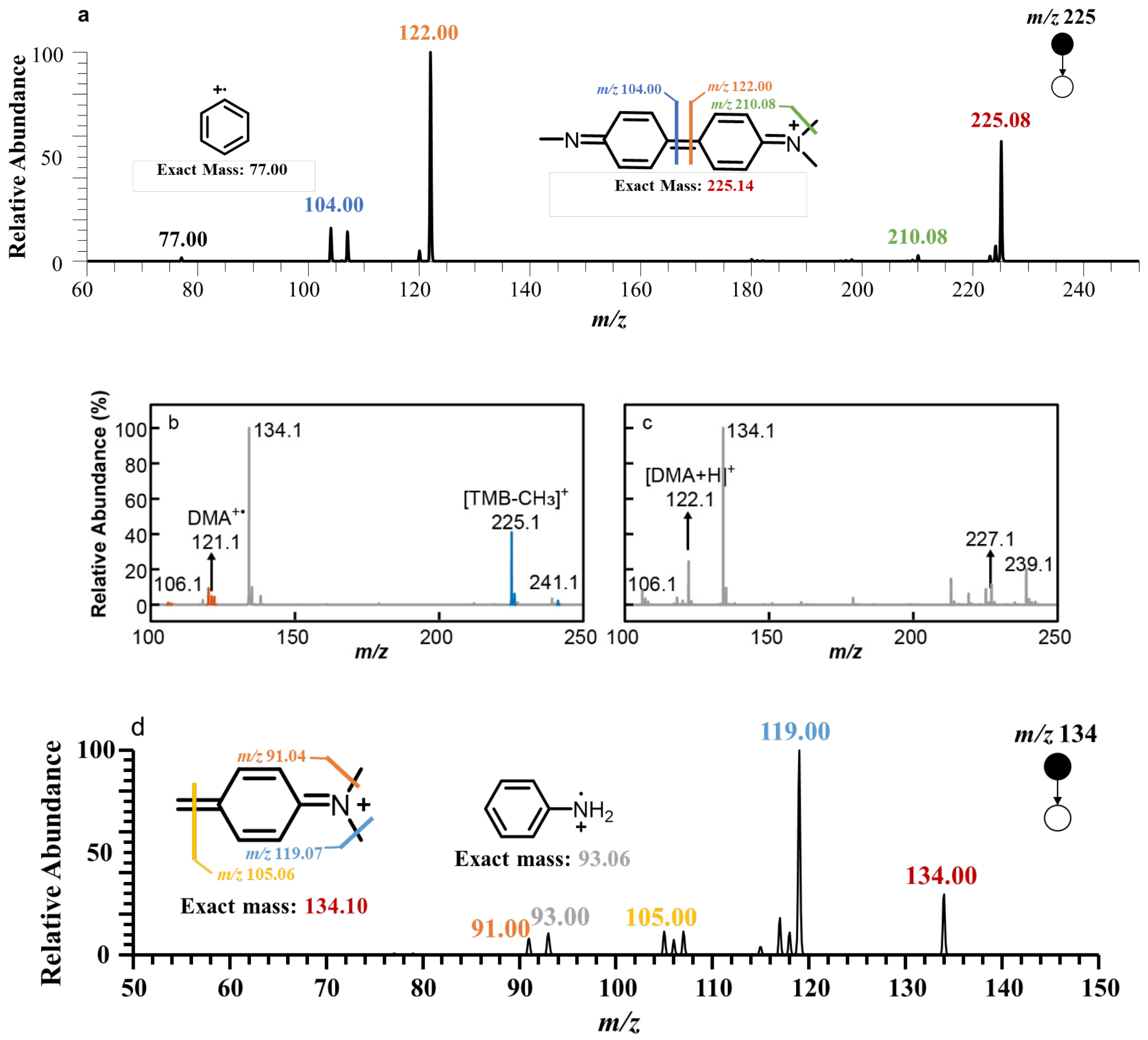

Figure S9. (a) Tandem MS for the identification of *m/z* 225.1 product obtained from the photooxidation of DMA in CH_2Br_2/Hexane with $CsPbBr_3$ NCs. (b) Mass spectrum of the reaction product from the mixture of DMA and $CsPbBr_3$ NCs in DBM/Hexane after 1 hour of 473 nm excitation. (c) Mass spectrum of the mixture of DMA and $CsPbBr_3$ NCs in DBM/Hexane without photoexcitation. (d) Tandem mass spectrum for identification of *m/z* 134.1 species.

The identification of *m/z* 225.1 species as the demethylated TMB ion (TMB-CH_3^+) was made via tandem MS shown in Fig. S6a. For this, collision-induced dissociation was implemented to observe the fragments from the *m/z* 225.1 peak. Two additional peaks at *m/z* 122.0 and *m/z* 104.0 in addition to *m/z* 225.1 are assigned to the two fragments resulting from the breaking of C=C bond between the two rings of TMB-CH_3^+ as illustrated in the figure. The mechanistic pathway is shown in Scheme 2 of the main article. Fig. S6b and c compare the mass spectra of the reaction products from the mixture of DMA and $CsPbBr_3$ NCs in DBM/Hexane with and without photoexcitation. *m/z* 225.1 species is seen only with photoexcitation. However, *m/z* 134.1 species appears in both conditions. The structure of this species is determined from the tandem MS as shown in Fig. S6d, which shows several fragments of *m/z* 134.1 species illustrated in the figure.

S[1] S. V. Rosokha, J. K. Kochi, *J. Am. Chem. Soc.* **2007**, *129*, 3683-3697.
